# Increasing vineyard sustainability: innovating a targeted chitosan-derived biocontrol solution to induce grapevine resistance against downy and powdery mildews

**DOI:** 10.3389/fpls.2024.1360254

**Published:** 2024-02-07

**Authors:** Daphnée Brulé, Marie-Claire Héloir, Thibault Roudaire, Jérémy Villette, Silvère Bonnet, Yoann Pascal, Benoît Darblade, Philippe Crozier, Philippe Hugueney, Véronique Coma, Benoit Poinssot

**Affiliations:** ^1^ UMR Agroécologie, INRAE, Institut Agro Dijon, Université de Bourgogne, Dijon, France; ^2^ Elicityl, Crolles, France; ^3^ Phyteurop, Paris, France; ^4^ UMR-A 1131 Santé de la Vigne et Qualité du Vin (SVQV), Université de Strasbourg, INRAE, Colmar, France; ^5^ Laboratoire de Chimie des Polymères Organiques, Université de Bordeaux, CNRS, Bordeaux INP, UMR 5629, Pessac, France

**Keywords:** *Vitis vinifera*, induced resistance, biocontrol product, chito-oligosaccharides, chitosan, degree of polymerization

## Abstract

The European Green Deal aims to reduce the pesticide use, notably by developing biocontrol products to protect crops from diseases. Indeed, the use of significant amounts of chemicals negatively impact the environment such as soil microbial biodiversity or groundwater quality, and human health. Grapevine (*Vitis vinifera*) was selected as one of the first targeted crop due to its economic importance and its dependence on fungicides to control the main damaging diseases worldwide: grey mold, downy and powdery mildews. Chitosan, a biopolymer extracted from crustacean exoskeletons, has been used as a biocontrol agent in many plant species, including grapevine, against a variety of cryptogamic diseases such as downy mildew (*Plasmopara viticola*), powdery mildew (*Erysiphe necator*) and grey mold (*Botrytis cinerea*). However, the precise molecular mechanisms underlying its mode of action remain unclear: is it a direct biopesticide effect or an indirect elicitation activity, or both? In this study, we investigated six chitosans with diverse degrees of polymerization (DP) ranging from low to high DP (12, 25, 33, 44, 100, and 470). We scrutinized their biological activities by evaluating both their antifungal properties and their abilities to induce grapevine immune responses. To investigate their elicitor activity, we analyzed their ability to induce MAPKs phosphorylation, the activation of defense genes and metabolite changes in grapevine. Our results indicate that the chitosans with a low DP are more effective in inducing grapevine defenses and possess the strongest biopesticide effect against *B. cinerea* and *P. viticola*. We identified chitosan with DP12 as the most efficient resistance inducer. Then, chitosan DP12 has been tested against downy and powdery mildews in the vineyard trials performed during the last three years. Results obtained indicated that a chitosan-based biocontrol product could be sufficiently efficient when the amount of pathogen inoculum is quite low and could be combined with only two fungicide treatments during whole season programs to obtain a good protection efficiency. On the whole, a chitosan-based biocontrol product could become an interesting alternative to meet the chemicals reduction targeted in sustainable viticulture.

## Introduction

Plants are in constant interaction with a variety of microorganisms, including pathogens like bacteria, fungi, oomycetes, and viruses. These crop pathogens significantly diminish the agricultural yields and product quality, resulting in substantial financial losses (Savary et al., 2019). To ensure both satisfactory yield and harvest quality, plant protection necessitates numerous chemical treatments, which can have detrimental impacts on the environment and human health. The present regulations in France and Europe aim to reduce the pesticide use in agriculture by 50% and phase out the most harmful substances by 2025 to help recover the Europe’s biodiversity by 2030.

Viticulture holds great agricultural and economic importance in many countries. However, the most commonly cultivated grapevine (*Vitis vinifera*) is highly susceptible to cryptogamic diseases like downy mildew (caused by the oomycete *Plasmopara viticola*), grey mold (*Botrytis cinerea*), and powdery mildew (*Erysiphe necator*). These destructive diseases frequently occur, impacting yield, wine flavor, and quality. Their control heavily relies on frequent fungicide applications. Beyond resistant cultivars, an interesting alternative involves stimulating the plant immune system with elicitors, natural molecules that mimic pathogen attacks. Plants possess the ability to detect various microbe-associated molecular patterns (MAMPs; Dodds and Rathjen, 2010) initiating diverse defense mechanisms. The recognition of these consistent microbial markers by pattern recognition receptors (PRRs; Boller and Felix, 2009; Boutrot and Zipfel, 2017) triggers plant defense responses (Jones and Dangl, 2006). These responses encompass generating reactive oxygen species (ROS), phosphorylating mitogen-activated protein kinases (MAPKs), synthesizing phytoalexins, and expressing defense-related genes (Yu et al., 2017).

In the last two decades, the significance of chito-oligosaccharides as novel biopesticides has garnered attention (Ait Barka et al., 2004; Aziz et al., 2006; Trotel-Aziz et al., 2006; Chandra et al., 2017). Among these compounds, chitosan has gained notable traction in plant protection as a natural fungicide and elicitor of plant immunity. This compound, a polycationic β-1,4-linked D-glucosamine biopolymer, is obtained through deacetylation of chitin derived from crustacean shells and fungal cell walls. Due to its biocompatibility, biosafety, biodegradability, and accessibility, chitosan finds widespread applications in many fields, ranging from food packaging to cosmetics, medical industry, and agriculture (Meynaud et al., 2023). The antifungal and antibacterial properties of chitosan against various pathogens are well-established, as it reduces infections in different crops like pea, grapevine, wheat, cucumber, tobacco and barley (Vander et al., 1998; Ben-Shalom et al., 2003; Aziz et al., 2006; Iriti et al., 2006; Faoro et al., 2008; Iriti and Varoni, 2015; Kheiri et al., 2016; Romanazzi et al., 2016).

In laboratory, chitosan triggers grapevine defense responses, including phytoalexin production, MAPKs phosphorylation, and defense gene expression, resulting in resistance against *B. cinerea* and *P. viticola* (Aziz et al., 2006; Brulé et al., 2019). While chitosan shows promising results under controlled conditions, its adoption in plant protection practices remains limited, likely due to its variable effectiveness in vineyards (Dagostin et al., 2011). Some studies have also highlighted chitosan’s effectiveness and utilization in postharvest decay control of fruits (Romanazzi et al., 2018; Duan et al., 2019). However, despite the numerous advantageous properties and agricultural applications of chitosan, the precise molecular mechanisms underlying its elicitation potential remain unclear. This lack of clarity hinders establishing a direct correlation between defense elicitation and plant protection. The biological impact of chitosan hinges on physicochemical attributes such as the deacetylation degree (DDA), and the molecular weight (MW), directly depending on the polymerization degree (DP). Notably, chitosan oligomers with lower MW have been proven more effective in inducing defense responses compared to higher MW counterparts (Lin et al., 2005; Aziz et al., 2006).

The aim of this study was to harness the complete phytosanitary potential of chitosan by using an optimized structure, with the aim of introducing a biocontrol product that surpasses the effectiveness of current solutions against grapevine downy and powdery mildews. Our investigation centered around six chitosan variants with diverse degrees of polymerization (DP) ranging from low to high (12, 25, 33, 44, 100, 470), all featuring a robust deacetylation degree (DDA) exceeding 93%. We scrutinized their biological activities by evaluating both their antifungal properties and their abilities to elicit grapevine immune responses in controlled settings and real vineyard conditions.

From these evaluations, we concluded that all chitosans do not possess the same biological activities. Thus, we identified the chitosan of DP12 as the most potent and comprehensively characterized candidate. We have also investigated the protection efficiency of combining chitosan with only two fungicide treatments, with the additional goal of reducing the excessive chemical applications.

## Materials and methods

### Plant and fungal materials

Grapevine (*V. vinifera* cv. Marselan) herbaceous cuttings were grown in a greenhouse until they had developed 6–8 leaves. The first and second youngest adult leaves from each plant were used for experiments, as previously indicated (Steimetz et al., 2012).

Grapevine samples (*V. vinifera* cv. Chardonnay, roodstock 3309C), collected from the Marsannay-la-Côte vineyard of the University of Burgundy (France), were used for *P. viticola* infection tests on leaf discs and qRT-PCR.

Grapevine downy mildew (*P. viticola*) was routinely maintained on *V. vinifera* cv. Marselan plants as previously described (Lemaître-Guillier et al., 2017).

The BMM strain of *B. cinerea* used (Zimmerli et al., 2001) was grown on Petri dishes containing V8 medium ½ diluted, KH_2_PO_4_ 5 g/L, agar 30 g/L, pH 6.0 for two weeks in the dark (22°C). Conidia were collected with water, filtered to remove mycelia, counted and kept at 4°C prior to infection assays.

### Chitosans and chemicals

Chitosans were provided by Elicityl (Crolles, France). Their origin is the exoskeletons of crustaceans. They were hydrolyzed, purified by chromatography and finally their degrees of polymerization (DP) and deacetylation degree (DDA) were evaluated by ^1^H NMR analysis (**Table 1**). The chitosan with the lowest DP was more deeply characterized in terms of molecular weight by size exclusion chromatography (SEC) and thermal resistance (according to Meynaud et al., 2023). Chitosans with low DPs (12 and 25) were dissolved in sterile ultrapure water and those with medium or high DPs (33, 44, 100, 470) were dissolved in acetic acid pH 4.5 (0.1%, 0.1%, 0.2% and 0.3% respectively).

**Table 1:** Characterization of the different chitosans.

Name	Deacetylation degree (DDA)	Polymerization degree (DP)	Polymerization degree (DP)
	(^1^H-RMN) ^a^	(^1^H-RMN) ^b^	(NDS)
**DP12**	98.2%	9.9	23
**DP25**	98.4%	28	25.2
**DP33**	97.8%	40	33
**DP44**	97.4%	78.1	44.7
**DP100**	93.4%	na	93/110
**DP470**	92.8%	na	~500

a- Deacetylation degrees from ^1^H-NMR (1H NMR 400 MHz (Bruker, Condition: chitosan (10 mg/ml) in D2O/DCl at 80°C, n=3).

b- Polymerization degree from ^1^H-NMR at room temperature in D20: DCl mixture (n=3).

NDS (3, 5 Dinitrosalicylic acid) is an acid reagent used for the determination of reducing sugars.

Chitooligosaccharides and Oligogalacturonides (COS-OGA) mixture solution has been used as a positive control with the homologated biocontrol product “BLASON” provided by Cerience. Chemicals provided by Phyteurop have also been used as positive controls in vineyard trials: the homologated copper mixture solution “Bouillie Bordelaise CAFFARO WG” and the sulfur solution “LUCIFERE” to protect grapevine against downy or powdery mildew, respectively.

### MAPKs activation

Discs of grapevine leaves from greenhouse cuttings were first vacuum-infiltrated with water, then floated on water (lower leaf surface facing the solution) during 3h before adding elicitor solutions. Discs were treated with the various chitosans (1 mg/mL), COS-OGA (62.5 mg/L) or water (as control) and harvested 20 min post-treatment. MAPKs activation was detected after immunoblotting of the extracted proteins using anti-p42/44-phospho-ERK antibody (Cell Signaling, Danvers, MA), as previously described (Brulé et al., 2019; De Bona et al., 2019). Transfer quality and homogeneous loading were checked by Ponceau red staining.

### Analysis of defense gene expression by quantitative polymerase chain reaction

For greenhouse assays, grapevine leaf discs were floated on water during 3h, then treated with the different chitosans (1 g/L), COS-OGA (62.5 mg/L) or water (as control) and harvested 3h post-treatment. Total RNA was extracted by using the Spectrum™ Plant Total RNA Kit (Sigma), according to the manufacturer’s instructions.

For vineyard grapevine assays, plants were sprayed with chitosans of different DPs (2 g/L), COS-OGA (125 mg/L) or control solutions with a knapsack sprayer and two youg adult leaves were harvested and frozen 10h post-treatment (hpt). RNA extraction was then carried out by the addition of TRIzol® (Invitrogen, Life Technologies, Saint-Aubin, France) following the manufacturer’s instructions.

For both, reverse transcription was performed using Superscript IV (Invitrogen) following the manufacturer’s protocol. Real-time qPCR was carried out as described previously (Trdá et al., 2014). The relative transcript level was calculated using the ΔΔCt method (Livak and Schmittgen, 2001) with the validated grapevine reference gene *VvVATP16* (Gamm et al., 2011) whose primers are described **Table 2**.

**Table 2:** List of the primers used.

Name		Forward Primer (5’->3’)	Reverse Primer (5’->3’)
*VATP16*	V-type proton ATPase 16kDa subunit	CTTCTCCTGTATGGGAGCTG	CCATAACAACTGGTACAATCGAC
*ROMT*	Resveratrol O-methyltransferase	TGCCTCTAGGCTCCTTCTAA	TTTGAAACCAAGCACTCAGA
*STS1.2*	Stilbene synthase	AGGAAGCAGCATTGAAGGCTC	TGCACCAGGCATTTCTACACC
*PR3.4C*	Endochitinase (*PR3)*	TCGAATGCGATGGTGGAAA	CGTCGCCCTAGCAAGTGAG

### Metabolomic analysis by LCMS

Leaf discs of grapevine cuttings grown in greenhouse were floated on water (lower leaf surface facing the solution) during 3h before adding elicitor solutions of the different chitosans (1 g/L), COS-OGA (62.5 mg/L) or water (as control). Twenty four h after treatment, discs were harvested, freeze-dried and ground in a mortar to obtain a fine powder. Thirty to 40 mg of powder was extracted with methanol (50 μL/dry mg) containing 5 μg/mL of chloramphenicol used as an internal standard. Analyses were performed on Dionex Ultimate 3000 ultra-high performance liquid chromatography (UHPLC) equipment coupled to a Thermo Exactive high-resolution mass spectrometer (HRMS). The analyses were carried out in positive and negative mode. The methods used have been previously described (Martin et al., 2021).

### *Botrytis cinerea* and downy mildew assays

For *B. cinerea* growth inhibition assays, the direct antifungal activity of chitosans was assessed by growing 270 µL of *B. cinerea* conidia (2.10^5^ c/mL) in Potato Dextrose Broth (PDB) 6 g/L, with 30 µL of different final concentrations of chitosans (25, 50, 250 and 500 mg/L), in a 100-wells microplate honeycomb Bioscreen. The growth of *B. cinerea* was followed by optical density at 492 nm using the Thermo Labsystem Bioscreen C system (cyan filter) with a reading every 2 hours during 60h (20°C, dark, continuous agitation).

For *P. viticola* infection on grapevine cuttings, the lower leaf surface was sprayed in greenhouse with elicitors (30 mg/L). Two days post-treatment (dpt), treated leaves were sprayed with a freshly prepared suspension of sporangia (2.10^4^ sp/mL) and plants were maintained in 100% relative humidity for 4h in darkness. Leaf discs were punched 5 dpi and transferred on moist Whatman paper in a plastic box maintained in 100% relative humidity under a 10/14h day/night cycle at 20/18°C. Infection intensity was assessed 7 dpi by measuring the sporulating area by using the image analysis Visilog 6.9 software (Kim Khiook et al., 2013).

For *P. viticola* infection on leaves harvested in vineyard 3 dpt, the leaf discs were punched in the laboratory, transferred on moist Whatman paper in a plastic box, inoculated and maintained in the same conditions as previously. Infection intensity was assessed 7 dpi by image analysis previously described.

For toxicity assays on *P. viticola* zoospores, a suspension of *P. viticola* sporangia (1.10^5^ sp/mL) was treated with different concentrations of chitosan. One hour and a half later, released zoospores, moving on a 1 mm^2^ square of a Malassez hemocytometer, were counted during one minute.

For *B. cinerea* infection on leaves harvested in vineyard 2 dpt, the leaf discs were punched in the laboratory, transferred on moist Whatman paper in a plastic box, inoculated on the upper surface with 1000 conidia in a 20 µL-droplet of 6 g/L PDB and maintained in the same conditions as previously. Infection intensity was quantified at 5 dpi by measuring the macerated lesion diameter.

### Vineyard experimental trials

The vineyard experimental trials have been realized between 2020 and 2023 on different grapevine cultivars (Merlot, Cabernet franc, Pinot Noir, Carignan, Chardonnay) in different locations (Nohic, Laruscade, Allones, Nimes, Cardet, Villevieille, Marsannay) to be representative of the different french vineyards. The experimental trials have been designed with four Fisher complet randomized blocks per modality. For downy mildew assays, the vineyards were brumerized with water and artificially inoculated. For powdery mildew assays, vineyard trials were realized in natural conditions. All product applications were realized preventively every 7 to 9 days during the entire period of grapevine susceptibility. Spraying was carried out with a knapsack sprayer (airblast) by treating each side of the grapevine row with a volume of slurry between 150 and 300 L/ha, depending on the vegetative stage of the plant and the spacing between rows in the vineyard plot.

Observations are made daily and complete notations are triggered when the disease is significantly present on the observed organs (leaves and clusters). In general, two to three notations on leaves and bunches are carried out between fruit set (BBCH 71) and the beginning of veraison (BBCH 81). The ratings consist of assessing the intensity (severity) of the disease on leaves or on clusters from a sample of 100 leaves or 50 clusters per elementary plot, i.e. 400 leaves and 200 clusters per modality. From the values observed in the untreated control plot, the protection efficiency of each modality is calculated for each block by the Abott formula (((untreated control – modality)/untreated control) x 100) followed by the mean efficacy +/- SE of the 4 blocks.

## Results

### The chitosan’s degree of polymerization influences its biopesticide effect

**Figure 1:**
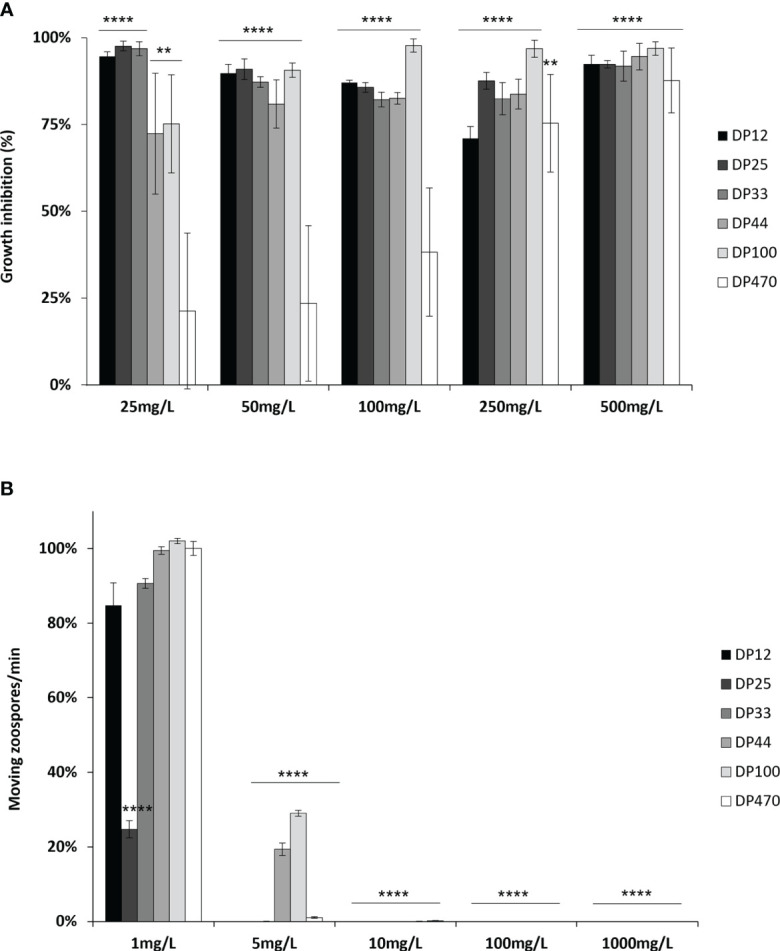
Biopesticide effects of chitosan with different DPs on *Botrytis cinerea* and *Plasmopara viticola.* **(A)** *B*. *cinerea* conidia were treated with increasing concentrations of chitosan DP12 to DP470 and mycelial growth was followed by optical density at 492 nm. Values represent the mean of growth inhibition ± standard error (SE) of triplicate data obtained in three independent experiments (n=9) at 60 hours and are expressed as a percentage of the control, set as 0%. Asterisks (*) indicate significant differences relative to the control using an unpaired heteroscedastic Student’s *t* test (**, P<0.01, ****, P<0.0001). **(B)** *P. viticola* sporangia were treated with increasing concentrations of chitosan DP12 to DP470 and released moving zoospores were counted for one minute on a 1 mm^2^ square of a Malassez hemocytometer. Values represent the mean ± SE of triplicate data obtained in three independent experiments (n=9) and are expressed as a percentage of the control, set as 100%. Asterisks indicate significant differences relative to the control using an unpaired heteroscedastic Student’s *t* test (****, P<0.0001).

A wide range of chitosans was used in this study (**Table 1**). The characterization of these chitosans based on their size and degree of deacetylation using various methods such as ^1^H NMR analysis and assessment of reducing sugars, revealed that the selected chitosans were nearly completely deacetylated. Moreover, their DP ranged from 10 to 500, thereby validating the earlier characterization of some among them (Huet et al., 2023). The direct biopesticide activity of the different chitosans were investigated on the necrotrophic fungus *B. cinerea* and the biotrophic oomycete *P. viticola*. The anti-*Botrytis* activity was determined by following the mycelial growth of *B. cinerea in vitro*. After a 60-hour treatment period, all the chitosans inhibited the growth of *B. cinerea* at low concentrations; however, variations were observed based on their DP. Chitosans DP12, 25 and 33 exhibited the highest fungitoxic effects, as they almost completely inhibited the growth of *B. cinerea* at the lowest concentration of 25 mg/L, with rates of inhibition reaching 95%, 98%, and 97%, respectively (**Figure 1A**). Chitosan DP44 and 100 showed an intermediate antifungal activity with 81% and 91% of growth inhibition at 50 mg/L while chitosan DP470 was more variable and less toxic on *B. cinerea*, and reached 88% of growth inhibition only at 500 mg/L (**Figure 1A**).

Their biopesticide activity was then assessed on the motility of *P. viticola* zoospores. All the different chitosans are toxic at very low concentrations as they totally inhibited the release and the motility of *P. viticola* zoospores from 1000 to 10 mg/L (**Figure 1B**). At 5 mg/L, chitosans DP12, 25, 33 and 470 always prevent the motility of zoospores while 19% and 29% moving zoospores are still observed after treatment with chitosans DP44 and 100, respectively. At 1 mg/L, there are as many moving zoospores as in the control for all the different chitosans, except for DP25, which is the most toxic. Although different bioactivities have been observed between chitosans depending on their DP, these results indicate a strong biocide effect of chitosans.

### Effects of the chitosan’s DP on the elicitation of grapevine immune responses

The importance of the chitosan’s DP for its elicitor activity was investigated on the grapevine immune responses such as MAPKs phosphorylation, defense gene expression and production of defense metabolites such as phytoalexins.

**Figure 2:**
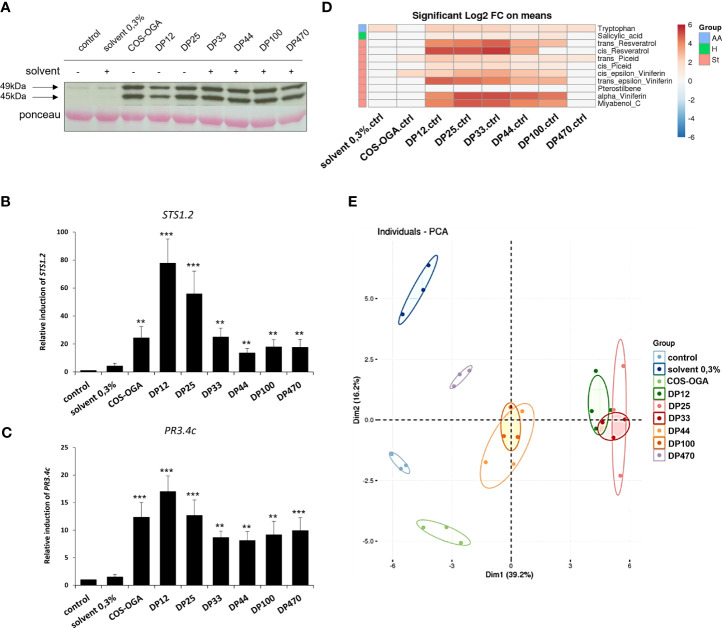
Defense response-induced with the different chitosans in grapevine. Leaf discs were floated on chitosan solutions (1g/L). Chitosan DP12 to DP470 were tested and compared to the negative control (water) and the previously characterized COS-OGA (62,5mg/L) as positive control. **(A)** Activation of two mitogen-activated protein kinases (MAPKs) in grapevine leaf discs, 20 min after elicitor treatment, detected by immunoblotting with α-pERK1/2 (Brulé et al., 2019). **(B, C)** Expression of defense genes encoding a stilbene synthase (*STS1.2*, **B**) and a chitinase (*PR3.4c*, **C**) measured by qPCR in grapevine leaf discs, 3h after treatment with the different chitosans. Values represent the mean ± SE of triplicate data obtained in three independant experiments (n=9). Asterisks indicate statistically significant differences between water and chitosan treatment, using an unpaired heteroscedastic Student’s *t* test (**, P<0.01, ***, P<0.001). **(D)** Log2 of significant metabolite fold changes 24 hpt. Indicated pairwise comparisons are given by shades of red or blue colors according to the scale bar. Metabolites were grouped according to their chemical family as amino acids (AA), hormones (H) and stilbenes (St). Data represent mean values of three biological replicates for each condition. Statistical analyses were performed using Tukey’s Honest Significant Difference method followed by a false discovery rate (FDR) correction, with FDR < 0.05. For FDR ≥ 0.05, Log2 fold changes were set to 0. **(E)** Global grapevine leaf disc metabolite changes 24 h after treatment with the different chitosans or control treatments. Principal component analysis (PCA) was performed on all quantified compounds in all conditions. Data represent mean values of three biological replicates for each treatment.

Similar to the positive control COS-OGA, a rapid phosphorylation of two MAPKs with relative molecular masses of 45 and 49 kDa, has been observed 20 min after treatment with the different chitosans, whatever their DP (**Figure 2A**). These 2 MAPKs were almost not activated in water- and solvent-treated control leaf discs (**Figure 2A**).

In response to the chitosans of different DP, the expression of defense genes known to be induced by different MAMPs in grapevine (Poinssot et al., 2003; Dubreuil-Maurizi et al., 2010; Trdá et al., 2014; Brulé et al., 2019) was quantified by qPCR. Three hours post-treatment (hpt), the positive control COS-OGA and all the chitosans markedly induced the expression of two selected defense genes encoding a stilbene synthase (*STS1.2* encoding the last enzyme of the stilbene phytoalexins pathway) and an acidic chitinase (*PR3.4c*). The results showed that chitosan DP12 was always the most active molecule (**Figures 2B, C**).

Metabolomic analyzes were then carried out 24 hpt and the heatmap presented **Figure 2D** indicates the compounds significantly different between the water control and the tested conditions. If the acidic solvent 0.3% (negative control) did not induce the production of defense compounds, the COS-OGA (positive control) slightly elicit the production of the two phytoalexins ε-viniferin and piceid at the concentration used and at the selected time point. The most marked significant differences concerned the production of stilbene phytoalexins such as resveratrol, α- and ε-viniferins, myabenol C and to a lesser extent piceid, the defense-related phytohormone salicylic acid, and the tryptophan amino acid (**Figure 2D**). Compared to water control, the differences at the level of stilbenes were strongly marked for chitosans DP12, 25 and 33, moderately for DP44 and 100, and low for DP470. These observations are corroborated by the principal component analysis (PCA, **Figure 2E**) which highlighted three groups related to the chitosan’s DP: highly elicitor (DP12, 25, 33), intermediate (DP44 and 100) and weakly active (DP470). COS-OGA and the acidic solvent 0.3% were separated from the control (**Figure 2E**).

Taken together, these results confirmed that chitosans with smaller DPs are most effective in eliciting grapevine immune responses in plants grown in greenhouse-controlled conditions.

### Effect of the chitosan’s DP on the induced resistance against downy mildew in grapevine plants grown in greenhouse

**Figure 3:**
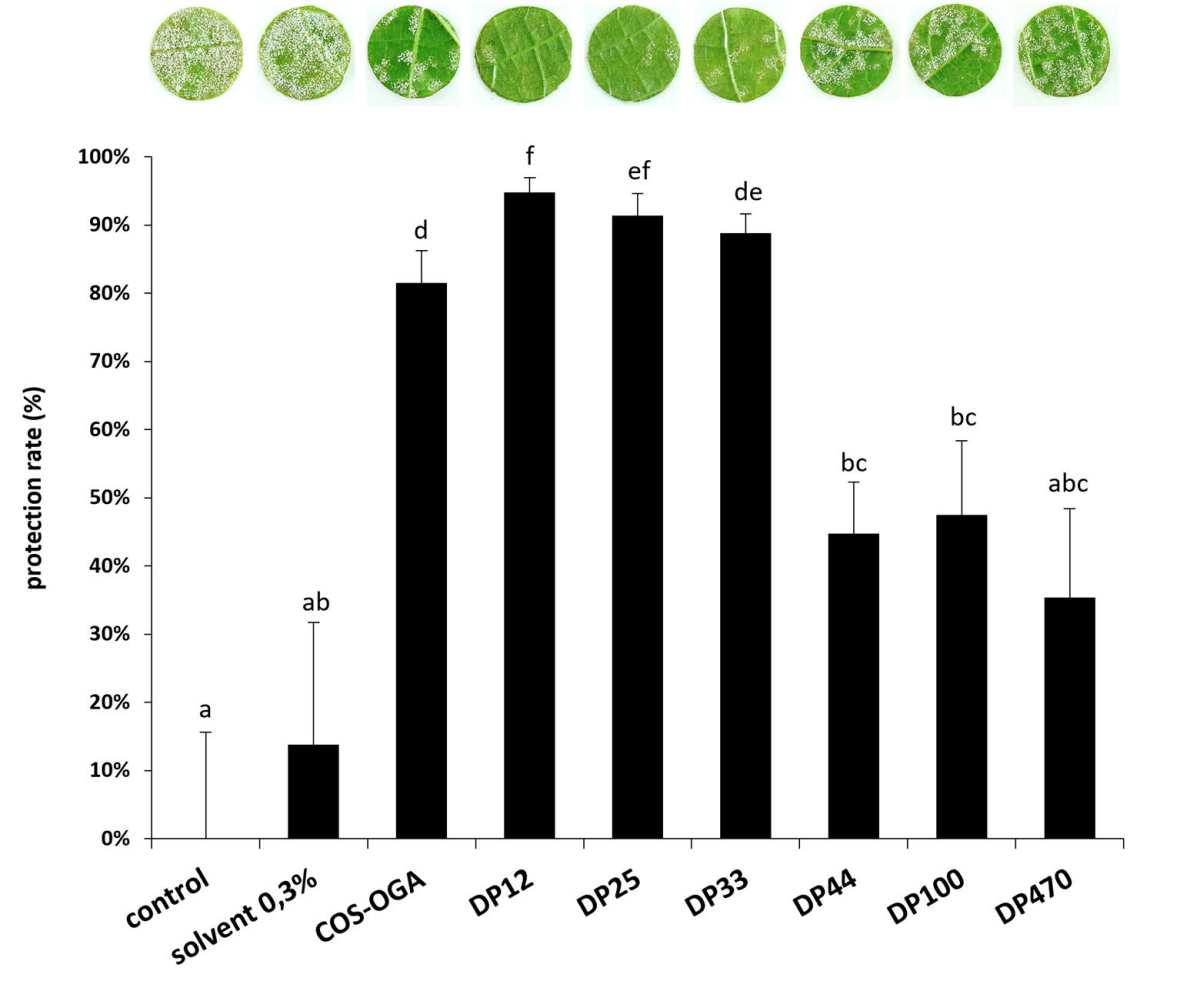
Chitosan-induced resistance against *Plasmopara viticola* under greenhouse conditions. Grapevine cuttings were sprayed with chitosan solutions (30 mg/L) 48 h before inoculation. Leaf discs were punched 5 dpi and the disease caused by *P. viticola* was assessed at 7 dpi. Sporulating leaf area was evaluated by image analysis Visilog 6.9 software (Kim Khiook et al., 2013). Values represent the mean of protection rate ± SE (n=36 discs from 3 different plants/condition) from one representative experiment out of three. Different letters indicate a statistically significant difference between treatments (Kruskal Wallis followed by Mann Whitney *post hoc* with P< 0 05).

The efficiency of the different chitosans to induce grapevine resistance was also investigated against downy mildew using a low dose of chitosan (30 mg/L). In greenhouse, grapevine leaves were treated with the different chitosans 48h prior to inoculation with *P. viticola*. Compared to the water control, showing a very important sporulation of *P. viticola* (0% protection), chitosan treatment with low DPs (12, 25 and 33) significantly reduced its sporulation with 95%, 91% and 89% of protection efficiency, respectively, while treatment with intermediate DPs (44 and 100) or high DP (470) only induced a partial resistance against downy mildew with respectively 45%, 47%, and 35% of protection (**Figure 3**).

### Chitosan with a low DP better elicits defense gene expression in grapevine plants from vineyard

**Figure 4:**
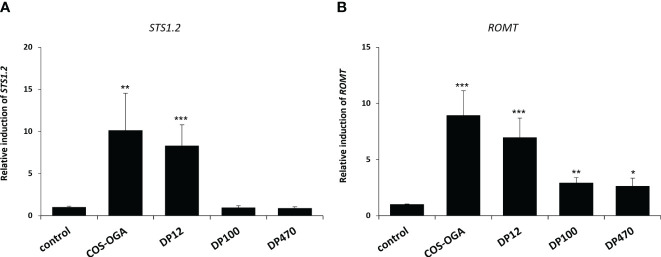
Chitosan-induced defense gene expression in grapevine leaves treated in vineyard. For both **(A, B)**, the expression of defense genes encoding a stilbene synthase (*STS1.2*) and a resveratrol O-methyltransferase (*ROMT*) measured by qPCR in grapevine leaves 10 h after being sprayed in vineyard. Values represent the mean ± SE of quadriplicate data (4 independent blocks) obtained in one experiment (n=4). Asterisks indicate statistically significant differences between water and chitosan treatment, using an unpaired heteroscedastic Student’s *t* test (*, P<0.05, **, P<0.01, ***, P<0.001).

The abilities of three chitosans with different DPs (12, 100 and 470) to induce defense gene expression was also investigated in grapevine plants grown in vineyard, 10h after treatment by using a manual knapsack sprayer. **Figure 4A** indicated that only chitosan DP12 and the positive control COS-OGA led to the expression of the defense gene *STS1.2* in grapevine leaves. **Figure 4B** showed that all chitosans significantly induced the expression of the defense gene *ROMT* which encodes the enzyme catalyzing the biosynthesis of pterostilbene from resveratrol. As previously shown on grapevine plants grown in greenhouse, among the chitosans with different DPs, DP12 was also the best elicitor in vineyard conditions. The fact that, in the same experiements and at the same timepoint (10 hpt), defense genes in different pathways such as *STS1.2* and *PR3.4C* were not induced in chitosan-treated young green berries (**Supplementary Figure S1**) suggests that the elicitation of plant immunity in leaves or fruits seems to be different, depending on the plant organ.

### Chitosan with a low DP better induces grapevine resistance against downy mildew in vineyard-treated plants

**Figure 5:**
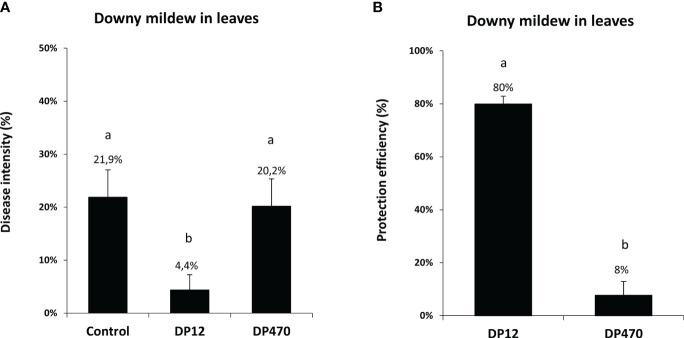
Comparison of the disease intensity and protection efficiency against downy mildew from grapevine leaves treated in vineyard with chitosan DP12 or DP470. Grapevine leaves from vineyard plants were sprayed with chitosan DP12 or DP470 (2 g/L) or untreated (control). Three days after treatment, leaves were harvested and leaf discs were punched and inoculated in the lab by *P. viticola* at 2.10^4^ sp./mL. **(A)** Disease intensity was quantified by measuring the sporulating area at 7 dpi with image analysis Visilog 6.9 software. Data represent the mean ± SE of quintuplicate data (5 blocks) obtained in three independent experiments (n=15) realized in 2020 in the experimental vineyard of Marsannay. **(B)** The protection efficiency against downy mildew has been calculated for chitosan DP12 vs DP470 using the Abott formula (see materials and methods). Different letters indicate significant differences between treatments using the Kruskal Wallis test followed by Dunn’s *post hoc* with P<0.05.

Thereafter, the ability of chitosans to trigger grapevine resistance against downy mildew was tested in vineyard, using one chitosan with the lowest DP (DP12) and one with the highest DP (DP470). Grapevine plants were treated in the vineyard using a knapsack sprayer with these two chitosans and then artificially inoculated in the lab with *P. viticola*. In vineyard-treated plants, the disease intensity of downy mildew was similar with DP470 (20.2%) compared to the untreated control (21.9%) whereas DP12 showed the lowest disease intensity of 4.4% (**Figure 5A**). Interestingly protection against downy mildew triggered by chitosan DP12 was higher than that obtained with DP470 and reached 80% or 8%, respectively (**Figure 5B**). In the same vineyard experiments, chitosan-treated leaves were also artificially inoculated in the lab with *B. cinerea*. Similarly to the results obtained with downy mildew, chitosan DP12 induced a better protection against gray mold than DP470 (**Supplementary Figure S2**). These data indicate that chitosans can induce grapevine resistance not only in greenhouse conditions but also in vineyard-treated plants. Altogether, these results led us to select chitosan DP12 as the most efficient resistance inducer to further evaluate its ability to protect grapevine against powdery and downy mildews in different vineyard conditions.

### Chitosan DP12 induces grapevine resistance against downy and powdery mildews in vineyard

**Figure 6:**
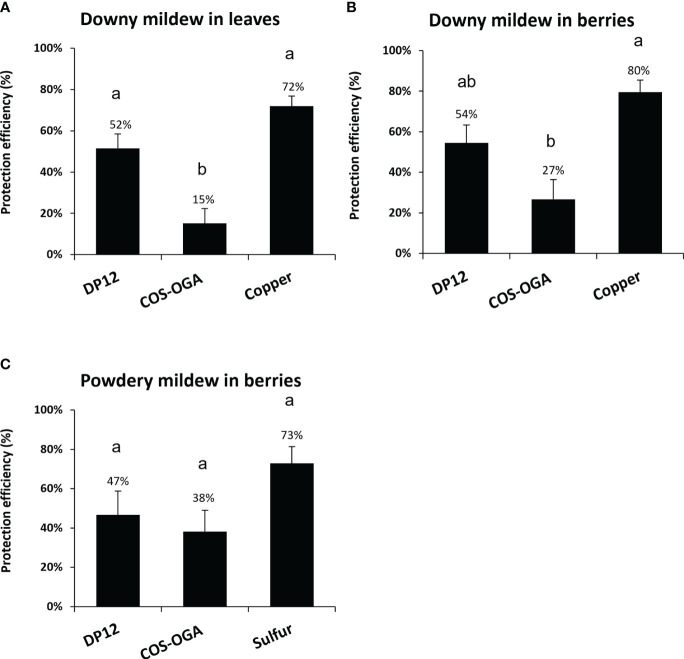
Protection efficiency of chitosan DP12 in grapevine leaves and berries against downy and powdery mildew in vineyard. Grapevine were sprayed with chitosan DP12 (400g/ha), COS-OGA (25g/ha; BLASON at 2 L/ha), copper mixture solution at 500g Cu/ha (Bouillie Bordelaise CAFFARO WG 20% Cu at 2,5 Kg/ha), sulfur solution at 2400g S/ha (LUCIFERE 3 L/ha at 800g S/L) or untreated (control). Protection efficiency of chitosan DP12 against downy mildew in leaves **(A)** and berries **(B)**, or against powdery mildew in berries **(C)**. Depending on the vineyard, between five to eight applications were carried out every 7 to 9 days and the protection efficiency was assessed 4 days after the last application between stages BBCH73 and 79. Values represent the mean of protection efficiency ± SE of quadriplicate data (4 blocks) obtained in three independent experiments (n=12) realized in 2023 in independent experimental vineyards on three different locations in France (i) (Nohic, Laruscade, Allonnes) on different grapevine cv (Cabernet franc, Merlot or Pinot noir, respectively) for downy mildew, and (ii) (Nîmes, Cardet and Villevieille) on different grapevine cv (Carignan, Chardonnay or Chardonnay, respectively) for powdery mildew. Different letters indicate significant differences between treatments using the Kruskal Wallis test followed by Dunn’s *post hoc* (P<0.05).

To evaluate the ability of chitosan DP12 to protect grapevine against downy mildew, experimental trials were performed in independent vineyards on three different locations in France (Nohic, Laruscade, Allones). Four modalities were tested: untreated control as negative control, treatment with the copper mixture solutionat 500g Cu/ha (Bouillie Bordelaise CAFFARO WG at 2.5 kg/ha with 200g Cu/kg) used as positive control, treatment with the homologated dose of the biocontrol product COS-OGA at 25g/ha (BLASON at 2L/ha with 12.5 g COS-OGA/L) and chitosan DP12 at 400 g/ha. Our results indicate that the mean protection efficiency quantified in grapevine leaves against downy mildew, between the physiological stages BBCH73 and BBCH79, was 15% with COS-OGA, 52% with chitosan DP12 and 72% with the copper mixture solution used as positive control (**Figure 6A**; **Supplementary Figure S3**). Similar results were obtained on grapevine berries with a protection efficiency of 27% for COS-OGA, 54% for chitosan DP12 and 80% for the copper mixture solution (**Figure 6B**; **Supplementary Figure S3**).

The efficiency of chitosan DP12 was also investigated to protect grapevine berries against powdery mildew in experimental trials realized in three independent vineyards on different locations in France (Nîmes, Cardet and Villevieille). Our results showed that the protection efficiency against powdery mildew was 38% with COS-OGA, 47% with chitosan DP12 and 73% with the sulfur treatment (Lucifere at 2400g S/ha) used as a positive control (**Figure 6C**; **Supplementary Figure S3**).

Interestingly these results were obtained in independent vineyards where the disease intensities in control plants were quite different (**Supplementary Figure S4**).

### Combination between chitosan DP12 and low amount of fungicides protects grapevine leaves and berries against downy mildew in vineyard

**Figure 7:**
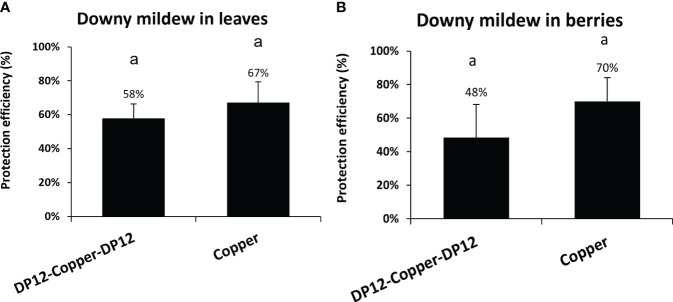
Protection efficiency of chitosan DP12 combined with low amount of fungicides in grapevine leaves **(A)** and berries **(B)** against downy mildew in vineyard. Ten applications were carried out every 7 to 9 days: either 4 times chitosan DP12 (400 g/ha) – 2 times copper mixture solution (Bouillie Bordelaise CAFFARO 500g Cu/ha) – 4 times chitosan DP12 (400 g/ha) or 10 times copper mixture solution (Bouillie Bordelaise CAFFARO) with 4 times (300g Cu/ha) – 2 times (500g Cu/ha) – 4 times (300g Cu/ha). The protection efficiency was assessed 4 days after the last application. Data represent the protection efficiency from one season program ± SE of quadriplicate data from 4 blocks (n=4) realized in 2021 in the French experimental vineyard of Allonnes on the Pinot noir cv. The annual amount of copper used was 1000g Cu/ha in the program “DP12-Copper-DP12” compared to 3400g Cu/ha in the “Copper” program. Similar letters indicate no significant differences between treatments using the Kruskal Wallis test followed by Dunn’s post hoc with P<0.05.

To evaluate the ability of chitosan DP12 to protect grapevine against downy mildew on a whole season program in vineyard, experimental trials were performed with 10 treatments per year. Three modalities were tested between physiological stages BBCH 35 to BBCH79: untreated control, 10 consecutive treatments with the copper mixture solution (Bouillie Bordelaise CAFFARO WG) with the following program: 4 times at 300g Cu/ha, 2 times at 500g Cu/ha at the flowering stage, and 4 times at 300g Cu/ha until veraison) used as positive control, or 4 treatments of chitosan DP12 (400 g/ha) followed by 2 treatments with the copper mixture solution (Bouillie Bordelaise CAFFARO WG at 500g Cu/ha) at the flowering stage, before the last 4 treatments with chitosan DP12 (400 g/ha). Our results indicated that the reduction of the chemical treatments by 80% (2 vs 10 treatments with the copper mixture solution) only decreases the protection efficiency from 67% to 58% on grapevine leaves (**Figure 7A**; **Supplementary Figure S5**) and from 70% to 48% on grapevine berries (**Figure 7B**; **Supplementary Figure S5**). Comparing the annual amount of copper used, 1000g Cu/ha were used in the program “DP12-Copper-DP12” compared to 3400g Cu/ha in the “Copper” program. Thus, the reduction by ~70% of the annual copper amount in the chitosan-based program (1000g Cu/ha vs 3400g Cu/ha) did not significantly decrease the protection efficiency (**Figure 7**).

## Discussion

Chitosan is well known for its antimicrobial and antifungal properties that can be used in plant protection (Ait Barka et al., 2004; Dagostin et al., 2011; Kheiri et al., 2016). This antimicrobial effect is largely influenced by the molecular weight, the degree of acetylation as well as the preparation methods used (Verlee et al., 2017). Despite the amount of literature available, the variety of chitosan used and their incomplete characterization make comparison almost impossible. In a previous work, we have shown that chitosan treatments lead to grapevine resistance against *Botrytis cinerea* and *Plasmopara viticola* in laboratory conditions (Brulé et al., 2019). However, the efficiency of chitosan to confer a good protection level in grapevine leaves in laboratory and greenhouse settings greatly varies compared to the results obtained in vineyard (Dagostin et al., 2011). The reasons for this discrepancy remain unclear but several hypotheses can be made: (i) all chitosans do not possess the same biological activity to induce grapevine resistance, (ii) the leaching of chitosan by rainfall, (iii) the photo-oxidation of chitosan due to sunlight exposure and (iv) its degradation by enzymes secreted by the microbial community of the phyllosphere. Recently, Meynaud et al. demonstrated that UV irradiation of chitosan is ineffective in degrading chitosan, indicating that the lower effectiveness of chitosan in open fields cannot be attributed to sunlight exposure (Meynaud et al., 2023).

In the present study, we showed that a well characterized chitosan, highly deacetylated with a low DP, is more efficient in protecting grapevine plants grown in greenhouse and vineyard, as previously demonstrated in laboratory conditions (Aziz et al., 2006; Younes et al., 2014). Additionally, it is interesting to note that the part of the elicitation and the anti-microbial effect in the biological activity of chitosan remain poorly reported in the literature and seems to be difficult to estimate. Our results suggest that elicitation of the plant immunity has a synergistic effect with the anti-microbial properties of chitosan, at least in laboratory and greenhouse conditions. The present data highlight the induction of defense genes in chitosan-treated leaves of grapevine plants grown under greenhouse as in vineyard, indicating that these defense genes could be used as molecular markers of the elicitation of plant immune responses. Interestingly, the tested defense genes were not induced at 10, 24 and 48 hpt (additional time points realized with no significant differences) in chitosan-treated young berries suggesting that the elicitation of the plant immunity could be different in leaves or fruits and thus could depend on the plant organ. So far, it might be interesting to discover the chitosan receptor to know its expression profile in the different plant organs. We have previously identified the two grapevine LysM receptor-like kinases VvLYK1-1 and VvLYK1-2 which participate in the chitosan perception (Brulé et al., 2019) whereas VvLYK5-1 or VvLYK5-2 are not involed (Roudaire et al., 2023). Interestingly, the expression of *VvLYK1-1* and *VvLYK 1-2* is quite low in the skin of young berries, suggesting that these co-receptors might be present in low amounts during the early development of this organ (Roudaire et al., 2023). Nevertheless, if the chitosan receptor is present in low quantity, we cannot exclude that these defense genes might be transiently induced at time points where samples were not harvested.

If the results obtained in the vineyard over several years were very interesting we also noticed that chitosan was less effective during rainy weather suggesting that chitosan might be rapidly leached. Thus, additional work is needed to find an appropriate formulation to still increase the protection efficiency of chitosan in vineyard conditions. Complementary trials conducted in different areas under real conditions have shown that chitosan efficiently protects grapevine leaves against downy or powdery mildew when the disease pressure is relatively low but that there was not a clear dose effect between 200, 300, 400 and 600 g/ha of chitosan. Thus, in a disease control program (against downy or powdery mildew), chitosan treatments can be inserted when the grapevine susceptibility to the disease is less strong, with a dose ranging from 200 to 400 g/ha depending on the quality of the spraying. When the disease pressure increases, the treatments with chitosan during the whole season with only two treatments of fungicides around the flowering stage offer a good vineyard protection to maintain the harvest. Finally, the association between chitosan and only two copper- or sulfur-based treatments during whole season programs could become an interesting alternative to greatly reduce the use of chemicals to improve the vineyards sustainability.

## Data availability statement

The original contributions presented in the study are included in the article/**Supplementary Material**. Further inquiries can be directed to the corresponding author.

## Author contributions

DB: Conceptualization, Data curation, Investigation, Methodology, Visualization, Writing – original draft. M-CH: Investigation, Methodology, Supervision, Writing – review & editing. TR: Investigation, Writing – review & editing. JV: Investigation, Writing – review & editing. SB: Data curation, Investigation, Methodology, Validation, Writing – review & editing. YP: Data curation, Investigation, Methodology, Writing – review & editing. BD: Data curation, Methodology, Supervision, Writing – review & editing. PC: Conceptualization, Investigation, Methodology, Supervision, Writing – review & editing. PH: Data curation, Formal analysis, Investigation, Methodology, Software, Supervision, Writing – review & editing. VC: Data curation, Investigation, Software, Supervision, Writing – review & editing. BP: Conceptualization, Funding acquisition, Investigation, Methodology, Project administration, Resources, Supervision, Validation, Writing – original draft, Writing – review & editing.
