# Supplementary material for: Increasing vineyard sustainability: innovating a targeted chitosan-derived biocontrol solution to induce grapevine resistance against downy and powdery mildews
Source: Front Plant Sci. 2024 Feb 7;15:1360254. doi: 10.3389/fpls.2024.1360254 (PMC10879612; doi:10.3389/fpls.2024.1360254)
Supplement: Supplementary file 1 [file DataSheet_1.pdf]

A **B**

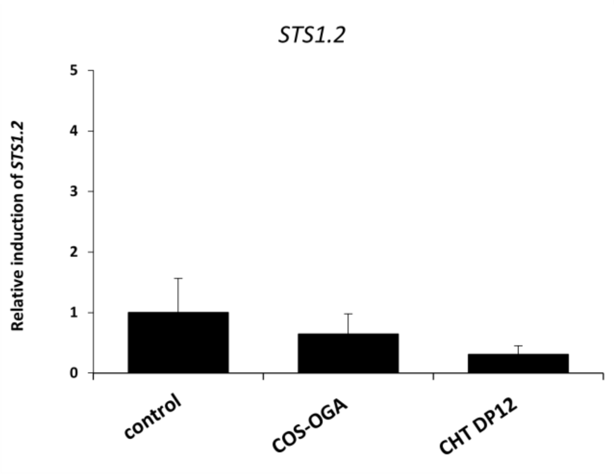

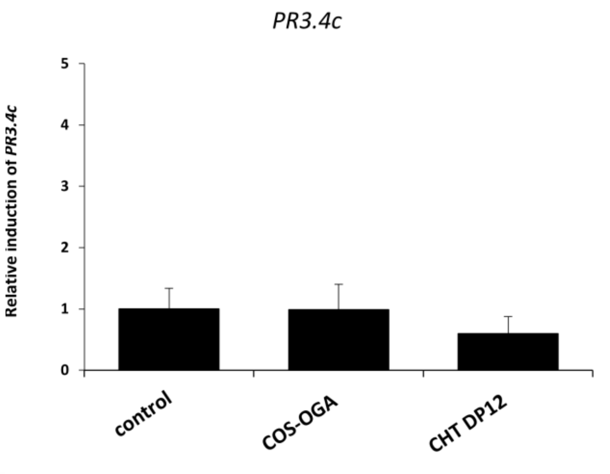

Figure S1. No induction of defense gene expression in young grapevine berries treated with chitosan DP12 in vineyard. For both A and B, the expression of defense genes encoding a stilbene synthase (*STS1.2*) and a chitinase (*PR3.4c*) measured by qPCR in grapevine berries 10 h after being sprayed in vineyard. Values represent the mean ± SE of quadriplicate data (4 blocks) obtained in one experiment (n=4).

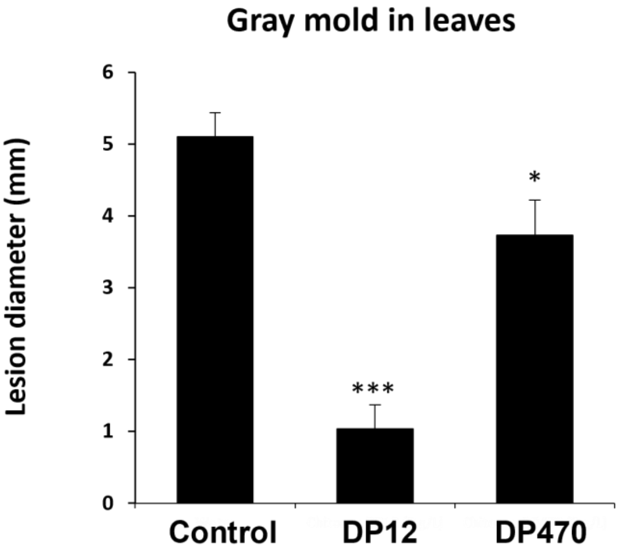

Figure S2. Comparison of the gray mold development in grapevine leaves treated with chitosan DP12 or DP470 in vineyard. Grapevine leaves from vineyard plants were sprayed with chitosan DP12 or DP470 (2 g/L) or untreated (control). Two days after treatment, leaves were harvested and leaf discs were punched and inoculated in the lab by *B. cinerea*. Conidia were prepared at 5.10^4 c/mL in 6 g/L PDB and one droplet of 20 µL (~1000 conidia) were deposited on each 2 cm-diameter leaf disc (abaxial side) previously placed on moistened Whatman paper. The infected plant material was then maintained in infection boxes in humid conditions under a day/night cycle 10/14 hours at 20/18°C before measuring the diameter of necrosis 5 dpi. Data represent the mean ± SE of quintuplicate data (5 blocks) obtained in one representative experiment out of three (n=5) realized in 2020 in the experimental vineyard of Marsannay. Asterisks indicate statistically significant differences between control and chitosan treatments, using an unpaired heteroscedastic Student's *t* test (*, $P<0.05$, **, $P<0.01$, ***, $P<0.001$).

A

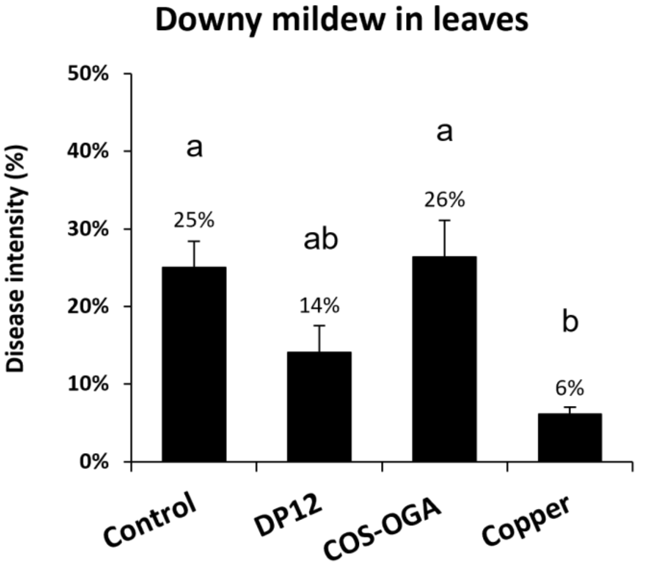

B

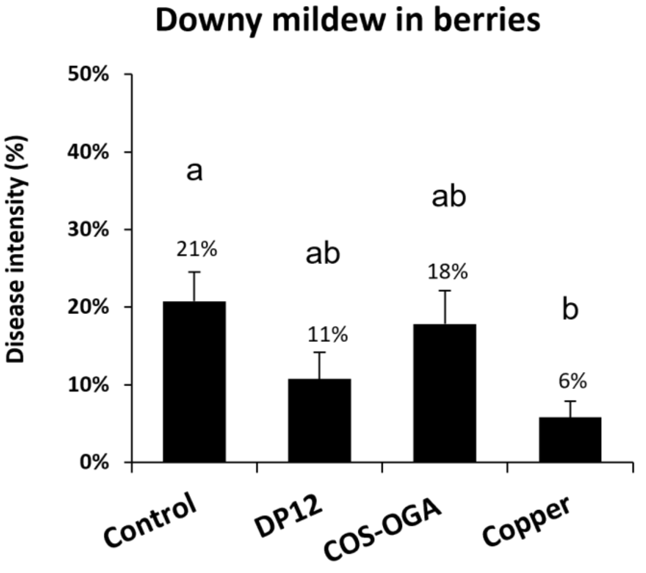

C

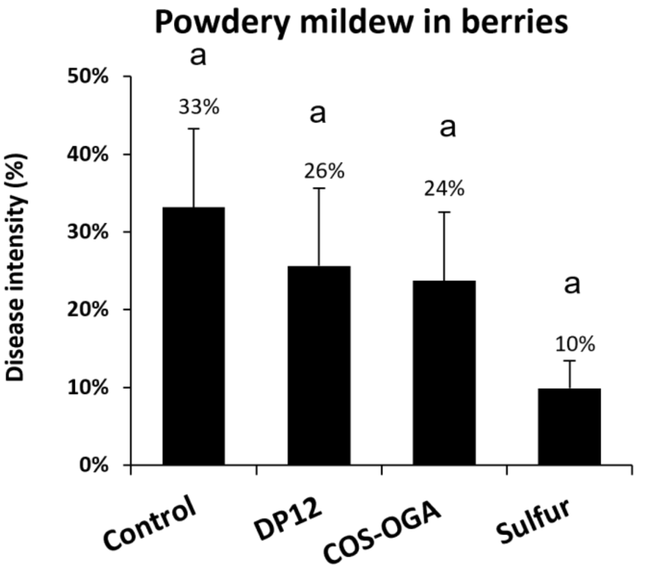

Figure S3. Disease intensities measured in vineyard to quantify the protection efficiencies of chitosan DP12 against downy or powdery mildew. Values represent the mean ± SE of quadriplicate data (4 blocks) obtained in three independent experiments (n=12). Data used to quantify the protection efficiency shown in Figure 6A (A), in Figure 6B (B) or in Figure 6C (C). Different letters indicate significant differences between treatment using the Kruskal Wallis test followed by Dunn’s post hoc with P< 0 05.

A

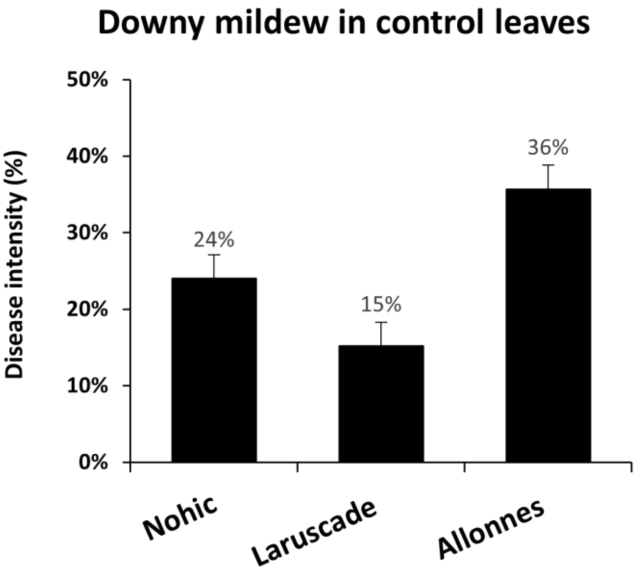

B

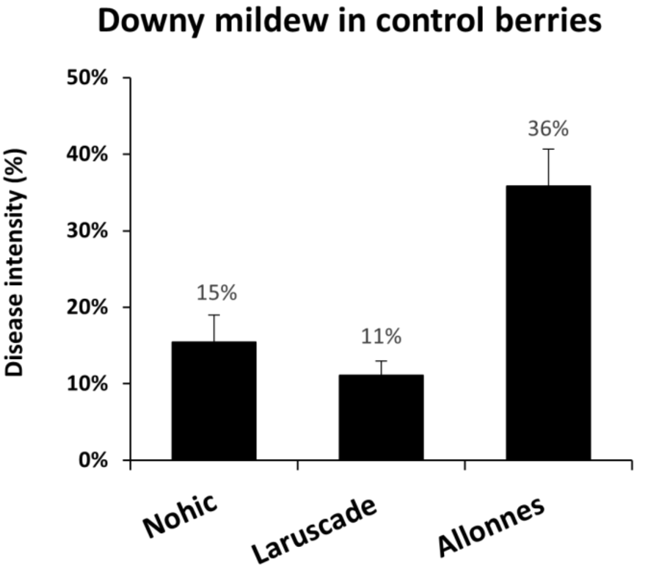

C

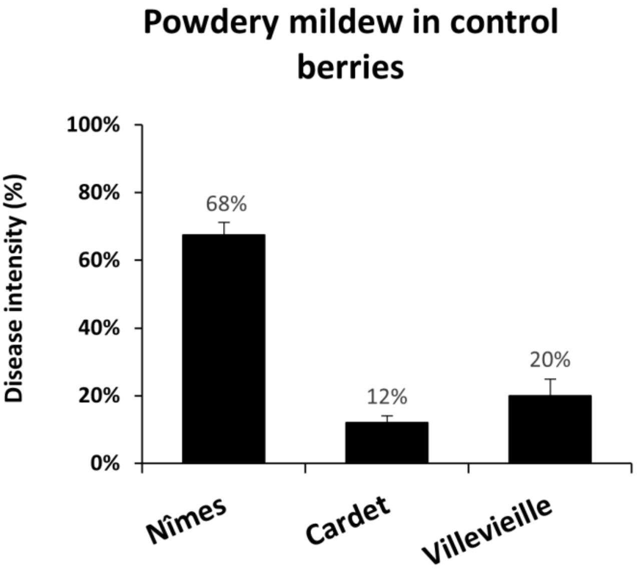

Figure S4. Disease intensities measured in each independent vineyard showing the basal pressure of downy or powdery mildew in control leaves and/or berries. Values represent the mean ± SE of quadriplicate data (4 blocks) obtained in one location (n=4). Data used to quantify the disease intensities of control shown in Figure S3A (A), in Figure S3B (B) or in Figure S3C (C).

A **B**

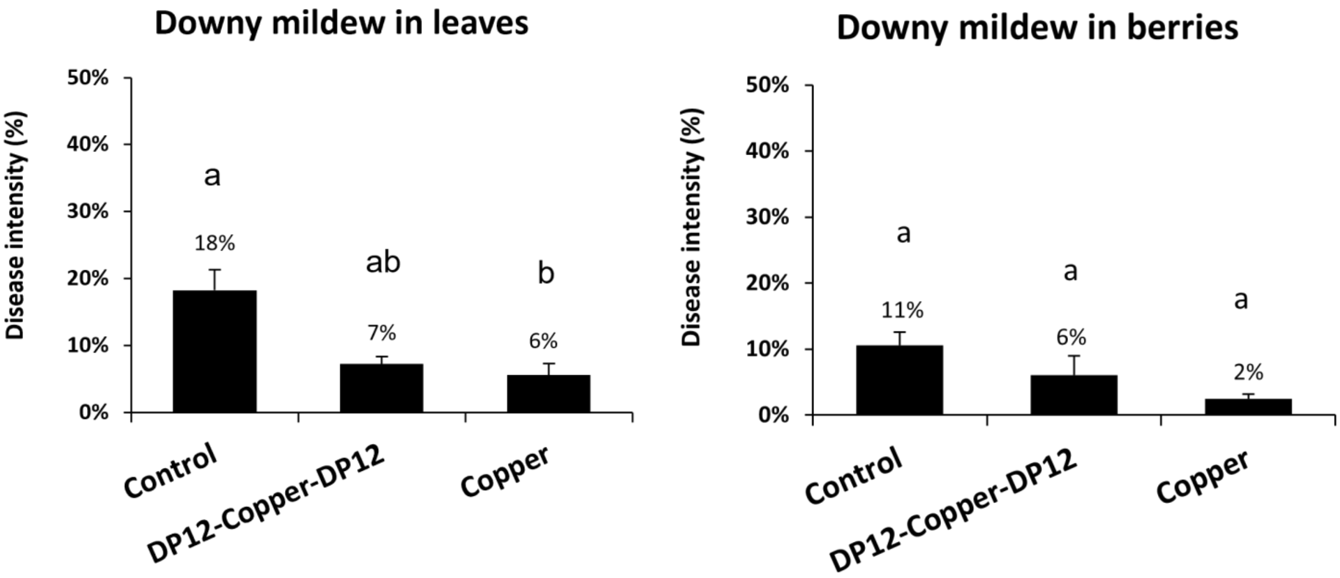

Figure S5. Disease intensities measured in vineyard programs to quantify the protection efficiencies of chitosan DP12 against downy mildew when decreasing the number of copper treatments. Values represent the protection efficiency from one season program ± SE of quadriplicate data from 4 blocks (n=4). Data used to quantify the protection efficiency shown in Figure 7A (A) or in Figure 7B (B). Different letters indicate significant differences between treatment using the Kruskal Wallis test followed by Dunn’s post hoc with P< 0 05.
